# Biomechanical Determinants of Ceiling-Tempo Double-Under Performance in World and Junior National Champions: Implications for Training and Injury Prevention

**DOI:** 10.3390/life16071078

**Published:** 2026-06-27

**Authors:** Kai Zhang, Yufeng Liu, Jianguo Kang, Qi Zhou, Xiuping Wang, Gongbing Shan

**Affiliations:** 1 Department of Physical Education, Xinzhou Normal University, Xinzhou 034000, China; xzsyzk@xztu.edu.cn (K.Z.); tyxkjg@xztu.edu.cn (J.K.); zhouqi@xztu.edu.cn (Q.Z.); wangxp@xztu.edu.cn (X.W.); 2 Biomechanics Lab, Faculty of Arts & Science, University of Lethbridge, Lethbridge, AB T1K 3M4, Canada

**Keywords:** temporal constraint, wrist–hand and ankle coordination, rate of force development, compact posture, rope–foot clearance heights, movement efficiency

## Abstract

Double-under jump rope performance requires rapid force production and precise coordination under extreme temporal constraints; however, the biomechanical determinants of elite high-speed performance remain unclear. This ceiling-performance biomechanical study examined speed-dependent motor control in world champions (*n* = 3, 19.7 years) and junior national champions (*n* = 5, 14.0 years) during double-under performance at 120 double-unders/min, 140 double-unders/min, and individualized ceiling tempo. Three-dimensional motion capture (300 Hz) synchronized with force plates (1500 Hz) quantified kinetic and kinematic adaptations across tempo conditions. Compared with junior national champions, world champions demonstrated shorter contact times, greater rates of force development, reduced center-of-gravity height and oscillation, more compact posture control with widened upper-limb positioning, smaller rope–foot clearance heights, and an increasingly ankle-dominant coordinative pattern between wrist–hand control and lower-limb movement under ceiling-tempo conditions. Collectively, these findings indicate distinct expertise-dependent differences in force-production, postural-control, and rope-coordination characteristics under ceiling-tempo conditions. In contrast, junior national champions demonstrated less pronounced temporal compression, maintained a comparatively extended posture, and appeared to approach a performance plateau beyond 140 double-unders/min. The findings provide biomechanical benchmarks for understanding ceiling-tempo performance and may inform training, movement retraining, and injury-prevention strategies in high-speed cyclic movement tasks.

## 1. Introduction

Jump rope has emerged as a global sport that uniquely integrates fitness accessibility with competitive intensity. Its widespread adoption stems from several practical advantages: minimal equipment requirements, adaptability to virtually any indoor or outdoor setting, and exceptional metabolic efficiency. Previous studies have demonstrated that regular jump rope training improves cardiorespiratory fitness, lower-limb power, balance, coordination, and bone health [1,2,3,4]. Consequently, the application of jump rope training has expanded across multiple domains, including adolescent physical health promotion, athletic conditioning for various sports, and rehabilitation protocols [5,6,7,8].

Although comprehensive global participation statistics remain limited, the scale of jump rope engagement in China reflects the sport’s growing international influence. According to the General Administration of Sport of China, more than 100 million individuals participate in jump rope activities nationwide [9]. Annual national finals regularly attract over 1000 athletes, while cumulative media exposure across television, newspapers, and digital platforms exceeds 200 million views [9]. Within such a large participation base, the demand for evidence-based biomechanical guidance for performance enhancement, training optimization, and injury prevention is increasingly evident.

Among jump rope techniques, the double under (DU) represents a key performance benchmark, requiring two complete rope revolutions within a single jump cycle [10]. Compared with the single under, this technique substantially increases temporal and coordinative demands by reducing the available time for rope clearance while requiring precise synchronization between upper- and lower-limb actions [11]. Successful execution depends on rapid wrist angular velocity generation, efficient regulation of whole-body motion, and accurate rope–foot timing under progressively increasing movement speeds. These characteristics make the DU an effective model for investigating speed-dependent motor control and movement regulation under conditions where performance efficiency and mechanical loading—and thus injury risk—are closely associated [7,11,12]. From an applied perspective, mastery of the DU also serves as a prerequisite for advanced multiple-under skills commonly performed in elite speed and freestyle competition [13].

Understanding how athletes adapt to increasing movement speed requires access to movement patterns reflecting fully developed skill execution [14,15,16]. Accordingly, a key methodological feature of this study is the use of ceiling-level data, representing coordination strategies emerging from long-term training rather than short-term experimental manipulation. In the present context, ceiling tempo represents the upper boundary of an athlete’s achievable DU performance capacity. Such data provide unique insight into optimized motor solutions in elite athletes [17,18,19], although they are inherently difficult to obtain due to limited access to high-level performers [20,21,22]. Importantly, the transition from sub-elite to elite performance requires multi-year, high-intensity training, and the resulting adaptations are reflected in measurable kinematic and kinetic differences. In the present study, world champions (WC, mean age: 19.67 years) and junior national champions (JNC, mean age: 14.00 years) were compared, with differences in training experience serving as indicators of accumulated motor learning and deliberate practice rather than direct evidence of causality. Quantifying these upper-performance characteristics provides practitioners with objective benchmarks for training progression, movement efficiency, load management, and injury prevention [23,24,25].

Despite the practical importance of speed manipulation in jump rope training, research examining motor control adaptations across progressively increasing DU tempos remains limited. Existing studies have primarily employed externally controlled tempo conditions without incorporating individual ceiling-tempo conditions [23,25], thereby limiting the ability to identify coordinative and biomechanical adaptations that emerge only when athletes approach their maximal-performance boundary. Given that movement speed is a major determinant of coordination demand and mechanical loading [12,26,27], identifying how athletes regulate movement across increasing tempos—including ceiling-level performance—is essential for bridging the gap between biomechanical evidence and applied coaching or rehabilitation practice.

Therefore, the present study aimed to examine speed-dependent motor control adaptations in WC and JNC performing DUs under progressively increasing and individualized tempo demands. Specifically, this study addressed two practitioner-oriented questions: (1) where do WC differ from JNC in skill control across increasing tempos, and (2) how does increasing movement intensity influence movement regulation within each group? By integrating kinematic and kinetic analyses across controlled- and ceiling-tempo conditions, this study sought to identify biomechanical determinants associated with elite performance and movement efficiency under increasing coordinative demand. These findings are intended to provide actionable, evidence-based insights for coaches, practitioners, and clinicians to support training optimization, injury prevention, and safe progression toward high-speed cyclic skill performance.

## 2. Materials and Methods

This study employed a cross-sectional tempo-escalation design to compare biomechanical characteristics between WC and JNC during double-under jump rope performance. All procedures were conducted in a controlled laboratory environment using synchronized three-dimensional motion capture and ground reaction force measurements. The protocol was designed to systematically increase movement intensity from controlled submaximal tempos to individualized ceiling-tempo conditions.

### 2.1. Subjects

To examine performance differences across expertise levels, eight male jump rope athletes from China were recruited and stratified into two groups based on competitive attainment: WC (*n* = 3, mean age 19.67 ± 2.08 years) and JNC (*n* = 5, mean age 14.00 ± 2.83 years). All participants were free from musculoskeletal injury in the six months preceding testing and refrained from strenuous physical activity for 24 h prior to data collection. Ethical approval was obtained from the institutional review board, and all participants provided written informed consent prior to participation.

Participant characteristics are presented in Table 1. As shown in Table 1, WC had accumulated approximately twice the training experience of JNC (11.33 vs. 5.33 years), reflecting the extended deliberate practice required to attain elite performance [17,24].

The ceiling-performance nature of elite biomechanics research inherently limits participant availability and sample size, particularly for genuine world-class performers capable of ceiling-tempo execution [25,28]. Nevertheless, such populations provide unique insight into the upper boundaries of human motor control developed through long-term deliberate practice.

### 2.2. Test Protocol

To examine motor control adaptations across increasing movement intensities, each participant completed a standardized testing session consisting of three tempo conditions. Following an individualized competitive-like ~15 min warm-up involving dynamic stretching and progressive-intensity rope skipping (target heart rate: 70–90% of personal maximum), participants performed DU trials at 120 DUs/min, 140 DUs/min, and an individualized ceiling-tempo condition.

Ceiling tempo was operationally defined as the highest tempo that could be successfully sustained while maintaining consecutive technically valid DUs. Participants were instructed to perform at their maximal achievable speed for a target duration of 10 s. The achieved performance was subsequently quantified from the recorded cycle times and represented each athlete’s individual ceiling-performance condition.

The first two conditions were externally regulated using a metronome and performed for 10 s each. In the ceiling-tempo condition, participants performed at their maximum achievable tempo, also with a target duration of 10 s; however, two JNC participants were unable to complete the full duration (7 and 8 s, respectively).

A minimum of 20 min recovery was provided between trials to minimize residual fatigue and ensure that performance reflected tempo-related coordination demands rather than accumulated physiological fatigue.

### 2.3. Three-Dimensional Motion Capture and Biomechanical Modeling

To quantify whole-body kinematics and rope motion across controlled and ceiling-tempo DU conditions, synchronized motion capture and force measurement systems were employed. Whole-body kinematics were recorded using a 13-camera VICON motion capture system (VICON V5, Oxford Metrics Ltd., Oxford, UK) sampling at 300 Hz, synchronized with four force plates (KISTLER, Model 9286BA, Winterthur, Switzerland) sampling at 1500 Hz. Prior to data collection, the motion-capture and force-plate systems were calibrated according to the manufacturers’ recommended procedures, achieving spatial accuracy of <1 mm and force measurement accuracy of <1 N.

**Figure 1:**
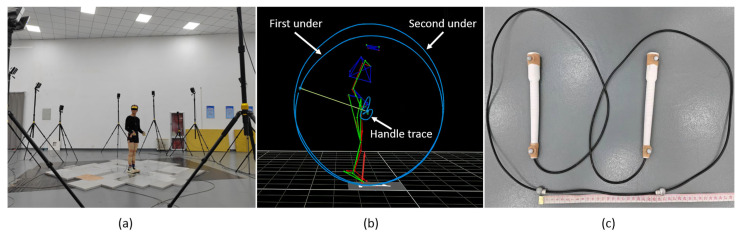
Experimental setup for three-dimensional motion capture during DU performance: (**a**) laboratory camera configuration, (**b**) representative synchronized 3D reconstruction of a DU cycle with corresponding ground reaction force (GRF) measurement from force plates, and (**c**) reflective marker configuration on the jump rope.

A total of 45 soft reflective markers (diameter = 9 mm) were applied: 39 markers positioned on anatomical landmarks of the participant’s body and 6 markers affixed to the rope [14,25]. Figure 1 illustrates the set-up of the motion data collection.

The 39 body markers were placed at specific anatomical locations, including the temporal and posterior head regions, sternoclavicular joint, xiphoid process, C7 and T10 vertebrae, scapulae, anterior and posterior superior iliac spines, acromioclavicular joints, lateral humeral epicondyles, radial and ulnar styloid processes, third metacarpal heads, lateral femoral condyles, tibial condyles, lateral malleoli, calcanei, and halluces. These marker coordinates were used to construct a validated 15-segment biomechanical model (head, upper and lower trunk, upper arms, lower arms, hands, thighs, shanks, and feet), which has been previously applied to complex sports skill analysis [29,30,31,32]. The inertial characteristics for each segment of the 15-segment model were determined by a statistical normal distribution [33,34].

Of the six rope markers, two reflective soft markers were positioned in the middle of the jump rope, spaced 25 cm apart (Figure 1c), to prevent interference with the feet during jumps. Placing two reflective markers in the middle of the rope serves two purposes: first, to mitigate the impact of rope midpoint rebound on data integrity; and second, to ensure comprehensive and accurate data collection of rope revolutions. Additionally, four markers, two on each handle, were utilized to delineate the rope during jumping.

The validated 15-segment biomechanical model was used to compute joint kinematics and whole-body center-of-gravity (COG) excursions across increasing tempo conditions. Kinematic and kinetic data were synchronized within the VICON system for subsequent analysis.

### 2.4. Data Processing and Parameter Selection

To extract biomechanically meaningful variables aligned with the study objectives, motion-capture and force data were processed and a set of functionally relevant biomechanical markers was selected to characterize movement coordination, tempo-control adaptations, and performance-related differences in a practitioner-accessible manner [14,25]. Raw motion capture data were filtered using a five-point smoothing algorithm within VICON Nexus software (version 2.8.1, VICON Motion Systems, Oxford Metrics Ltd., Oxford, UK), yielding three-dimensional coordinates for all markers.

To improve representation of stable movement patterns, multiple technically successful double-under cycles were extracted from each participant under each tempo condition. Because repeated cycles originated from the same athlete, they should not be considered fully independent observations. The repeated-cycle approach was adopted to improve the characterization of stable individual performance patterns and reduce the influence of cycle-to-cycle variability in this rare elite-performance population. Consequently, the statistical analyses should be interpreted as exploratory assessments of biomechanical patterns within this elite-performance cohort rather than as population-level estimates.

For each controlled tempo condition (120 and 140 DUs/min), five consecutive technically stable DU cycles were selected at approximately 1, 4, and 7 s, resulting in a total of 15 analyzed cycles per participant. For the individualized ceiling-tempo condition, due to inter-individual differences in successfully sustained performance duration (two JNCs failed to complete the 10 s test), 15 technically stable double-under cycles were extracted from the middle portion of the successfully completed trial. This procedure generated a tempo-based dataset representing progressively increasing coordinative and mechanical demands across controlled and maximal-performance conditions. Accordingly, a total of 45 DU cycles per participant were included for statistical analysis.

Consistent with the study objectives, previous biomechanical findings and fundamental principles of kinesiology research reporting [14,35,36], a focused set of functionally relevant variables was selected to characterize speed-dependent motor-control adaptations and performance-level differences in a biomechanically interpretable and practitioner-accessible manner:

- Tempo-control variables: cycle time and foot contact time;
- Whole-body and segmental motion variables: vertical ROM (range of motion) of COG and feet, and joint ROM (hip, knee, ankle);
- Clearance-related variables: toe height at foot-to-rope cross (i.e., toe clearance height);
- Kinetic variables: max vertical GRF, GRF increase rate and jump impulse.

In addition, two newly defined practitioner-oriented proxy parameters were introduced to enhance the applied interpretability of complex coordinative and postural-control adaptations:

- Upper-limb control proxy: mediolateral distance between rope handles, reflecting upper-limb coordination during DUs;
- Whole-body compression proxy: vertical distance between the head and toes, representing whole-body vertical compression primarily resulting from coordinated hip, knee, and ankle flexion–extension during DUs.

These variables were selected according to their functional relevance to double-under performance. Specifically, COG height and COG ROM were used to characterize vertical movement economy and temporal efficiency; rope-clearance height and rope midpoint trajectory were selected to quantify rope-control precision and spatial constraints; and the practitioner-oriented upper-limb control proxy and whole-body compression proxy were introduced to capture postural and coordinative adaptations that are readily interpretable by coaches and athletes. Together, these variables provide a concise yet functionally comprehensive representation of the kinetic, kinematic, temporal, and coordinative characteristics most relevant to high-speed DU expertise and ceiling-tempo performance.

To minimize the influence of anthropometric differences, all length-related variables were normalized to body height and all kinetic variables to body weight, thereby converting absolute measures into relative values and enabling more accurate between-group comparisons [37,38,39].

### 2.5. Statistical Analysis

To address the study objectives in a clear and practitioner-oriented manner, statistical analyses were structured around between-group comparisons and within-group tempo-dependent adaptations. Descriptive statistics (mean ± SD) were calculated for the selected variables. Data normality was assessed using the Shapiro–Wilk test.

Between-group differences (WC vs. JNC) at each tempo condition were examined using independent-samples *t*-tests (or Mann–Whitney U tests where appropriate). In addition to *p*-values, standardized effect sizes (Cohen’s d) were calculated to quantify the magnitude of between-group differences.

Within-group tempo-dependent adaptations were analyzed separately for each group (WC and JNC) using one-way repeated-measures ANOVA across the three tempo conditions (120 DUs/min, 140 DUs/min, and ceiling tempo). When significant main effects were identified, post hoc comparisons were performed using Tukey’s HSD test. The obtained *p*-values are reported where appropriate to facilitate the interpretation of tempo-dependent effects.

All analyses were conducted using IBM SPSS Statistics (Version 23.0), with nominal significance set at α = 0.05.

## 3. Results

The results are presented in three sections: (1) the coordinative and mechanical characteristics defining double-under skill uniqueness, (2) biomechanical differences between world champions and junior national champions, and (3) within-group tempo-dependent adaptations across increasing tempo conditions.

### 3.1. Skill Uniqueness

**Figure 2:**
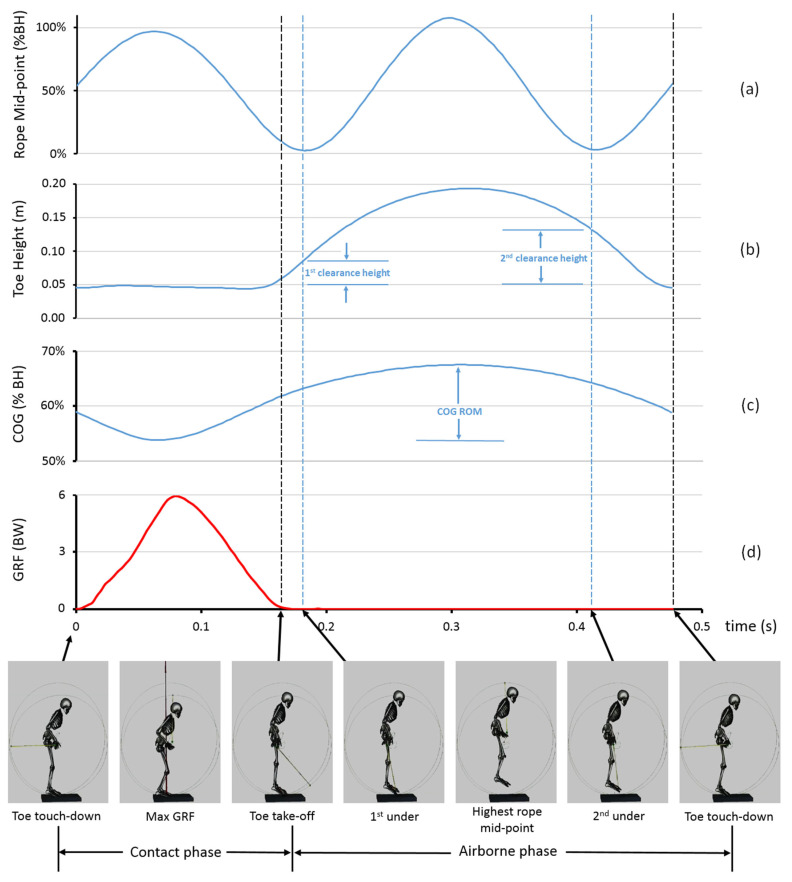
A representative double-under dataset obtained through synchronized measurements and biomechanical modeling. The figure illustrates the main characteristics of body–rope–ground interactions (graphs) for selected key biomechanical markers: (**a**) rope midpoint trajectory normalized to body height; (**b**) toe height during rope passage; (**c**) center-of-gravity (COG) height normalized to body height; and (**d**) ground reaction force (GRF) normalized to body weight. The lower panel illustrates whole-body joint coordination quantified using biomechanical modeling. GRF (BW): Ground reaction force normalized to body weight; COG ROM: Center-of-gravity’s range of motion in vertical direction.

Figure 2 presents a representative double-under cycle with synchronized measurements of selected key biomechanical markers, including rope midpoint trajectory, toe height, COG displacement, and GRF, together with the corresponding 15-segment biomechanical model and key temporal events during the movement cycle.

The double-under consisted of a short contact phase followed by a substantially longer airborne phase, with the airborne duration being approximately twice that of ground contact (Figure 2d). Within the airborne phase, the first rope under occurred immediately after take-off (interval between the black and blue dotted lines in Figure 2), whereas the second under occurred shortly before landing (interval between the blue and black dotted lines in Figure 2), demonstrating the extreme temporal compression required for successful double-under execution.

Distinct differences were observed between the two rope-clearance events. The first under exhibited a smaller rope–foot clearance height than the second under (Figure 2a,b), indicating that the first rope passage imposed greater coordinative and temporal demands. This reduced clearance window required highly precise synchronization between wrist–hand control and lower-limb movement immediately after take-off.

The synchronized COG and GRF profiles further revealed that vertical body motion during the double-under consisted of two mechanically distinct control patterns (Figure 2c,d). During the contact phase, vertical COG displacement was primarily generated through coordinated hip, knee, and ankle flexion–extension actions associated with force production and take-off preparation. In contrast, during the airborne phase, the body behaved as a relatively rigid projectile system with quasi-stable segmental alignment while the rope completed two revolutions beneath the feet (Figure 2, bottom panel).

These findings illustrate the unique coordinative structure of the double-under, in which explosive lower-limb force generation must be tightly integrated with rapid upper-limb rope control within an extremely restricted temporal–spatial window.

### 3.2. Between-Group Comparisons—Differences Between World and Junior National Champions

To identify the biomechanical determinants distinguishing elite from developing athletes, between-group comparisons were first conducted across the controlled and ceiling-tempo conditions.

Table 2 and Table 3 present the kinetic and kinematic characteristics of WC and JNC across 120 DUs/min, 140 DUs/min and individual ceiling-tempo performance. Table 4 summarizes the statistical significance and probability of finding effects for between-group comparisons at each tempo.

At the controlled tempos (120 and 140 DUs/min), both groups demonstrated generally similar movement patterns; however, several biomechanical markers consistently distinguished WC from JNC. Significant between-group differences (*p* < 0.05) indicated that WC exhibited greater maximal GRF and shorter foot contact times (except at 120 DUs/min) than JNC, whereas highly significant differences (*p* < 0.01) showed smaller first-under rope-clearance heights, lower peak rope midpoint positions, and greater whole-body compression in WC. These findings indicate more compact posture control and more precise rope–foot coordination during DUs in WC.

These between-group differences became more pronounced under ceiling conditions. Statistical analysis confirmed highly significant differences for all selected biomechanical markers (*p* ≤ 0.01; Table 4), with effect sizes indicating large between-group differences. Specifically, compared with JNC, WC demonstrated markedly lower COG height and COG ROM, a more compressed body posture with greater lateral arm opening, reduced rope clearance height, shorter cycle and foot contact times, and lower jump impulse, while simultaneously exhibiting substantially greater maximal GRF and rate of force development (RFD; from zero to maximum [40,41,42]).

Collectively, these findings indicate that WC adopted a compact, low-trajectory, and temporally compressed control strategy during ceiling-tempo performance, whereas JNC relied on a comparatively higher and less mechanically efficient movement pattern with larger safety margins and greater vertical displacement.

### 3.3. Within-Group Tempo-Dependent Adaptations

Following the identification of between-group differences, within-group analyses were conducted to examine how increasing tempo influenced kinetic and kinematic adaptations in WC and JNC.

#### 3.3.1. Kinetic Adaptations Across Increasing Tempo Conditions

Maximum vertical GRF remained relatively stable across tempo conditions in both groups. In WC, no significant difference was observed between the two controlled tempos (5.89–5.92 BW, *p* > 0.05), whereas a significant reduction occurred from 140 DUs/min to ceiling tempo (5.92 vs. 5.57 BW, *p* < 0.05) (Table 2 and Table 5). A similar pattern was observed in JNC, with stable max GRF values across controlled conditions (5.55–5.58 BW) followed by a significant reduction at ceiling tempo (5.58 vs. 5.31 BW, *p* < 0.05, probability = 0.80) (Table 3 and Table 5).

In contrast, the RFD demonstrated clear tempo-dependent divergence between groups. WC showed progressive and highly significant increases across all tempo transitions, from 74.92 BW/s at 120 DUs/min to 92.26 BW/s at 140 DUs/min and 133.56 BW/s during ceiling-tempo performance (all *p* < 0.01). JNC also demonstrated significant increases from 120 to 140 DUs/min (72.48 vs. 90.46 BW/s, *p* < 0.01); however, no further increase was observed under maximal-tempo conditions (90.46 vs. 91.87 BW/s, *p* > 0.05).

Jump impulse demonstrated an opposite trend to RFD. In WC, impulse decreased significantly across all tempo transitions, from 4.86 Ns/kg at 120 DUs/min to 4.26 Ns/kg at 140 DUs/min and 2.95 Ns/kg under ceiling conditions (all *p* < 0.01). JNC showed a similar progressive reduction, from 4.81 to 4.19 to 3.57 Ns/kg (all *p* < 0.01).

Both cycle time and foot contact time decreased progressively with increasing tempo in both groups, with WC exhibiting substantially greater reductions under ceiling-tempo conditions. In WC, cycle time decreased from 500 to 428 and 255 ms, while foot contact time decreased from 184 to 145 and 93 ms across the three tempo conditions (all *p* < 0.01). In JNC, cycle time decreased from 499 to 428 and 362 ms, whereas foot contact time decreased from 186 to 149 and 126 ms (all *p* < 0.01).

Collectively, these findings indicate that WC adopted a progressively more explosive and temporally compressed force-production strategy as movement speed approached ceiling tempo, whereas JNC appeared to reach a performance plateau beyond 140 DUs/min, particularly in RFD capacity.

#### 3.3.2. Kinematic Adaptations and Tempo-Dependent Postural Control

Kinematic adaptations accompanying tempo escalation are presented in Table 2 and Table 3, with Table 5 summarizing the statistical significance of within-group comparisons across tempo conditions.

As tempo increased, WC maintained relatively stable COG height across the controlled tempos, followed by a marked reduction under ceiling conditions, whereas COG ROM decreased progressively across all tempo conditions. In contrast, JNC demonstrated comparatively smaller tempo-dependent reductions in both variables. Specifically, WC COG height remained stable between 120 and 140 DUs/min (~68% vs. ~67% of BH, *p* > 0.05), but decreased substantially at ceiling tempo (~56% of BH, *p* < 0.01; Table 5). WC COG ROM progressively decreased from ~14% of BH at 120 DUs/min to ~12% at 140 DUs/min, and further to ~5% under ceiling conditions (all *p* < 0.01). The marked reduction in COG ROM together with the extremely short cycle time under ceiling tempo indicates that movement execution became increasingly ankle-dominant, with the hip and knee joints remaining relatively constrained to maintain a compact posture for maximal-speed performance. In contrast, JNC demonstrated only modest reductions in COG height and ROM under ceiling conditions (65.4% and 9.3% of BH, respectively). These findings indicate that WC adopted a lower and more vertically efficient posture under maximal demand, whereas JNC maintained greater vertical displacement.

**Figure 3:**
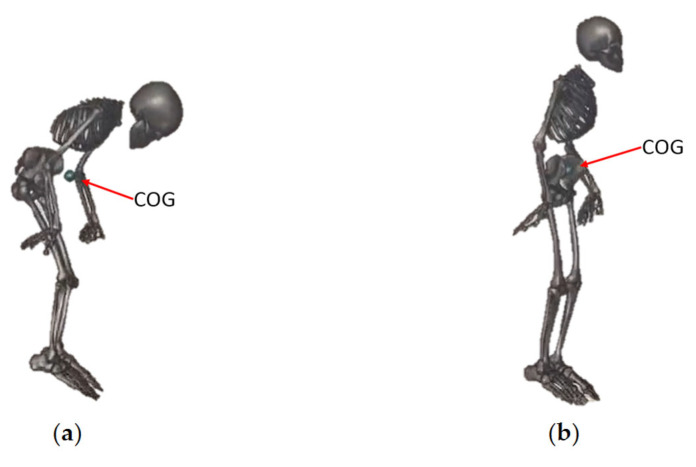
Typical posture during ceiling-tempo performance. (**a**) WC, (**b**) JNC.

The posture data further confirm distinct postural adaptations in WC as temporal and spatial constraints increased. Under ceiling conditions, WC demonstrated significantly greater upper-limb lateral opening (from ~45% at 140 DUs/min to ~53% of BH at ceiling tempo, *p* < 0.01) together with substantially greater whole-body compression (from ~87% to ~65% of BH, *p* < 0.01), reflecting widened upper-limb positioning and a more compact movement posture during performance (Table 5, Figure 3a). In contrast, JNC showed consistent posture across tempo conditions, with no significant tempo-dependent changes in either upper-limb control proxy (47.8–47.2% of BH) or whole-body compression proxy (90.8–90.7% of BH; Table 5, Figure 3b).

WC demonstrated pronounced reductions in rope–foot clearance height across all tempo conditions, particularly under ceiling conditions, whereas JNC exhibited more modest tempo-dependent adaptations. In WC, first-under clearance height decreased significantly from ~5 cm at 120 DUs/min to only 3 cm at ceiling tempo (*p* < 0.01; Table 5), while second-under clearance decreased from ~9 cm to ~4 cm (all *p* < 0.01). Although JNC also demonstrated reduced clearance heights, their values remained substantially greater under ceiling conditions (first under ~4 cm; second ~6 cm), indicating larger safety margins and less precise rope–foot coordination.

Both groups showed a similar adaptation pattern in highest rope mid-point control: relatively stable height from 120 to 140 DUs/min (*p* > 0.05), followed by a decrease beyond 140 DUs/min (all *p* < 0.01; Table 5). However, WC demonstrated a marked reduction in peak rope height from ~106% of BH at 140 DUs/min to ~102% at ceiling tempo (*p* < 0.01), whereas JNC maintained relatively elevated rope trajectories around ~106% of BH at ceiling tempo (Table 5). These findings indicate that the widened upper-limb positioning and more compact vertical posture of WC maintained a lower and more energy-efficient rope trajectory under maximal-speed conditions.

Collectively, these findings indicate that, under ceiling-tempo conditions, WC accommodated increasing tempo demands through reduced vertical displacement, minimized rope clearance margins, and dynamic postural adjustments. Movement execution became increasingly ankle-dominant, with rope control coordinated primarily between wrist–hand rotation and rapid ankle flexion–extension, while the hip and knee joints remained comparatively constrained to preserve the compact posture required for maximal-speed performance. In contrast, JNC did not demonstrate the same degree of temporal compression or postural adaptation under ceiling conditions.

## 4. Discussion

The present study examined tempo-dependent biomechanical adaptations in world champions and junior national champions during double-under performance under controlled and ceiling-tempo conditions. Consistent with the study objectives, the findings identified clear expertise-dependent differences in kinetic, kinematic, and coordinative control strategies, particularly under maximal temporal constraints. The results further revealed distinct coordinative and biomechanical solutions adopted under ceiling-tempo conditions. The following discussion addresses the biomechanical mechanisms underlying these adaptations, their practitioner and injury-prevention implications, and the methodological significance of the ceiling-performance research paradigm.

### 4.1. Decoding Ceiling-Tempo Performance of World Champions

The present findings indicate that ceiling-tempo double-under performance requires a fundamentally different coordinative solution characterized by explosive force production, temporally compressed movement execution, and highly precise rope–body synchronization. These expertise-dependent adaptations became particularly evident as movement tempo approached individual ceiling performance.

From a kinetic perspective, increasing tempo progressively shifted movement execution toward rapid force production within increasingly shortened contact durations. Although maximum vertical GRF decreased slightly under ceiling-tempo conditions in both groups, this reduction likely reflected the combined effects of shortened ground-contact time and reduced vertical displacement at maximal speed. More importantly, WC demonstrated a markedly greater ability to increase RFD as temporal constraints tightened, whereas JNC appeared to reach a plateau beyond 140 DUs/min. Simultaneously, WC exhibited substantially lower jump impulse under ceiling conditions, indicating more temporally efficient force production achieved within a reduced movement window [14]. Together with the pronounced reductions in cycle time and feet contact time, these findings indicate that WC relied on a highly explosive and temporally compressed movement strategy during ceiling-tempo performance.

The kinematic and posture-control findings further revealed that ceiling-tempo execution required an integrated coordinative adaptation within an increasingly restricted temporal–spatial window. As cycle and contact times decreased, WC progressively reduced both COG height and COG ROM, thereby minimizing unnecessary vertical displacement and shortening airborne duration. Simultaneously, the observed kinematic and kinetic patterns suggest that movement execution became increasingly ankle-dominant, with rapid ankle flexion–extension serving as the primary lower-limb control mechanism, while the hip and knee joints remained comparatively constrained to preserve a compact movement posture. These adaptations enabled faster and more temporally efficient movement execution under maximal-speed conditions.

To accommodate the progressively reduced spatial and temporal margins, WC additionally demonstrated distinct postural adaptations characterized by widened upper-limb positioning together with substantial whole-body compression. This coordinative configuration likely facilitated the maintenance of rope speed and trajectory while stabilizing the compact posture required for high-tempo execution. Consequently, WC were able to maintain extremely small rope–foot clearance margins, with first-under clearance reduced to approximately 3 cm under ceiling conditions. The lower rope trajectory and minimized vertical oscillation are consistent with a more mechanically and temporally efficient movement strategy optimized for increasing skipping tempo while preserving rope-control precision.

The present findings are generally consistent with previous double-under biomechanics research. Kim et al. [23] reported that skilled performers exhibited shorter movement durations and reduced vertical center-of-mass displacement than unskilled performers. The present study extends these observations by demonstrating that such characteristics become increasingly pronounced as performance approaches individualized ceiling tempo. In particular, the marked reductions in COG height, COG ROM, and cycle duration observed in WC suggest that minimizing vertical motion and maximizing temporal efficiency represent key adaptations for high-speed double-under execution. The present findings also complement the work of Bruce et al. [11], who demonstrated that double-under performance is sensitive to technique-related coordination changes under demanding conditions. Together, these studies support the view that successful high-speed double-under performance depends on highly refined coordinative adaptations rather than solely on physical capacity.

In contrast, JNC did not demonstrate the same degree of temporal compression, ankle-dominant coordination, or postural adaptation under maximal conditions. Instead, they maintained a comparatively extended posture associated with greater vertical displacement, higher rope trajectories, and larger rope–feet clearance heights. Although these adjustments may provide a larger safety margin for rope passage, they also increase airborne duration, movement time, and the mechanical demands associated with repeated high-speed performance.

Collectively, these findings indicate that the ability to increase ceiling double-under tempo depended on a temporally and mechanically efficient coordinative strategy characterized by explosive ankle-dominant force production, compact posture control, widened upper-limb positioning, and highly precise wrist–hand and ankle coordination within extremely restricted temporal–spatial margins.

### 4.2. Training Implications for the Transition to World-Class Performance

The present findings provide practical insight into how developing athletes may progress toward world-class double-under performance. From an applied perspective, the identified ceiling-tempo biomechanical characteristics may serve as objective benchmarks for skill optimization, movement retraining, load management, and the development of potentially safer and more mechanically efficient high-speed cyclic movement training programs.

Importantly, the findings suggest that JNC approached a performance plateau at approximately 140 DUs/min, particularly in their limited ability to further increase RFD and temporally compress movement execution. In contrast, WC continued to demonstrate substantial reductions in contact time, COG displacement, and rope-clearance height as tempo approached ceiling conditions. These observations indicate that the transition from junior elite to world-class performance likely requires additional years of deliberate practice focused on developing the coordinative and explosive-control adaptations necessary for maintaining movement efficiency under extreme temporal constraints. This study provides expert-modeled focused instruction that could lead to more effective learning compared with traditional training methods lacking such insights [43,44,45].

From a training perspective, three key adaptation targets emerged from the present findings. First, training should emphasize improving rapid ankle plantarflexion force-production capacity [46,47], particularly the explosive RFD characteristics of the gastrocnemius–Achilles tendon complex [48], which appeared central to successful ceiling-tempo execution. Second, postural-control training should facilitate the transition from a comparatively upright posture with arms positioned close to the body toward a more trunk forward-inclined and vertically compressed posture with widened upper-limb positioning [14,49]. This posture likely shifts the COG anteriorly beyond the base of support, thereby increasing dynamic balance demands during high-speed execution. Third, training should progressively enhance wrist–hand and ankle coordination under increasing tempo conditions [44,45] to improve rope-control precision within increasingly restricted temporal–spatial margins.

Collectively, these findings suggest that progression toward world-class double-under performance is not solely dependent on greater physical capacity but also on the development of a fundamentally different coordinative control strategy optimized for explosive and temporally constrained movement execution.

### 4.3. Injury-Prevention Implications for High-Speed Double-Under Training

While the biomechanical adaptations observed in WC enhanced movement efficiency and ceiling-tempo capacity, they may also increase mechanical loading on specific musculoskeletal structures [11,50]. The combination of forward trunk inclination, reduced lower-limb excursion, rigid proximal-joint stabilization, and rapid ankle-dominant force production likely places substantial repetitive stress on the ankle joint and associated tendon–muscle complexes during prolonged high-speed training. These findings therefore provide clinically and practically relevant insight for coaches, practitioners, and clinicians involved in high-speed jump rope training, return-to-sport progression, and long-term load management.

A particularly important injury-prevention implication was the extremely small rope–foot clearance employed by WC under ceiling conditions, with first-under clearance reduced to approximately 3 cm. Although this highly compact strategy improved movement efficiency and compact rope-clearance control, it also substantially reduced the movement safety margin and likely increased the technical demands placed on skill control precision and ankle loading during repetitive high-speed training. Achieving such precise rope clearance safely likely requires extensive long-term training involving thousands of repetitive movement cycles to progressively develop the necessary coordinative accuracy, ankle explosiveness, and dynamic postural stability. Consequently, developing athletes should avoid directly imitating the highly challenging elite clearance value at early training stages [44,45]. Instead, coaches should emphasize the gradual reduction in rope-clearance margins together with progressive improvements in temporal control precision, ankle force-production capacity, and movement stability to reduce overload risk during high-frequency repetitive training [14,25].

The present findings further suggest that excessive vertical displacement during repetitive high-frequency movements may increase lower-limb mechanical loading and energetic demand, consistent with previous observations in cyclic jumping tasks [14]. Accordingly, reducing COG height and vertical oscillation may simultaneously improve movement efficiency and decrease repetitive loading stress. These variables may therefore serve as practical biomechanical indicators for movement retraining and return-to-sport progression [51,52,53].

The ankle-dominant strategy observed in WC additionally highlights the importance of plantar-flexor strength and explosive force-production capacity for injury-risk management during repetitive high-speed training. Previous research identified reduced plantar-flexor strength as an intrinsic risk factor for Achilles tendon overuse injury [54,55]. The substantially greater RFD capacity demonstrated by WC suggests that the ability to generate timely explosive ankle force may contribute not only to maximal performance, but also to safer repetitive loading under extreme temporal constraints [56].

Collectively, the present results indicate that progression toward world-class double-under performance requires a careful balance between movement efficiency and mechanical loading management. The biomechanical benchmarks identified in this study may therefore assist coaches, clinicians, and practitioners in designing evidence-based training, rehabilitation, and injury-risk management programs for high-speed cyclic movements.

### 4.4. Ceiling-Performance Research Paradigm: Methodological Contributions, Limitations, and Future Directions

The present study contributes to a growing body of research highlighting the scientific value of ceiling-performance data in human movement analysis. Unlike conventional experimental approaches based primarily on externally imposed submaximal tempo conditions [25,57,58], the ceiling-tempo paradigm captured each athlete’s individually determined maximal-performance capacity. Whereas JNC demonstrated evidence suggesting a performance plateau beyond 140 DUs/min, WC continued to exhibit substantial coordinative and biomechanical adaptations as performance approached individualized ceiling tempo. This contrast highlights the added value of ceiling-performance analysis for identifying expertise-dependent characteristics that may not be apparent under standardized submaximal conditions. In addition, the selected and practitioner-oriented proxy parameters were introduced to facilitate biomechanically interpretable assessment of postural-control and coordinative adaptations under ceiling-tempo conditions. These variables were intentionally limited to biomechanical markers with direct performance and coaching relevance. Rather than reporting a large number of isolated kinematic variables, the present study focused on markers capable of characterizing temporal efficiency, postural adaptation, rope-control precision, and explosive force production, thereby facilitating interpretation by both researchers and practitioners. Together, these methodological features enabled identification of coordinative and biomechanical adaptations—particularly the highly compact and coordinatively demanding control strategies observed in WC—that may not emerge under standardized but non-maximal conditions. Consistent with previous arguments by Cavanagh and Hinrichs [20] and Shan [22], ceiling-performance data provide unique insight into the upper boundaries of human motor control and movement optimization consistent with adaptations associated with long-term deliberate practice.

At the same time, the ceiling-performance paradigm inherently imposes methodological constraints. The limited availability of WC athletes resulted in very small and unbalanced sample (WC *n* = 3; JNC *n* = 5). In addition, the repeated-cycle design resulted in multiple observations being obtained from the same athlete. Although this approach improved characterization of stable movement patterns and reduced the influence of cycle-to-cycle variability, within-athlete observations are not fully independent. These factors restrict statistical generalizability and should be considered when interpreting the magnitude of between-group differences. However, such limitations are common in elite-performance biomechanics research because athletes capable of performing at genuine world-class ceiling levels are rare [20,21,22]. Consequently, the present results should be interpreted as evidence from a rare elite-performance cohort rather than as directly generalizable estimates of the broader jump rope population. Nevertheless, large effect sizes and consistent patterns across synchronized kinetic and kinematic variables were observed. Future investigations involving larger elite cohorts may further refine the biomechanical benchmarks and coordinative principles identified in the present study.

Several additional limitations should also be acknowledged. First, the cross-sectional design does not permit causal determination of whether the identified biomechanical characteristics resulted primarily from long-term training adaptations, maturation-related influences, or pre-existing performer-specific attributes [59,60,61]. Because the world champions were older than the junior national champions, some observed differences may reflect developmental-stage influences in addition to expertise-related adaptations. Longitudinal investigations tracking athletes progressing from junior elite to world-class levels would provide valuable insight into the developmental emergence of ceiling-tempo control strategies. Second, the present study examined only male world champions, which limits the generalizability of the findings to female jump rope performers. Future research should investigate whether similar ceiling-tempo coordinative adaptations are present in female world-class performers. Finally, future studies integrating electromyography and fatigue-related neuromuscular responses may further clarify the physiological mechanisms underlying ankle-dominant explosive control during maximal-speed cyclic movement tasks.

Despite these limitations, the present findings demonstrate that ceiling-performance analysis can provide translationally meaningful biomechanical benchmarks relevant to sports biomechanics, physiotherapy, movement retraining, and injury-risk management. The ceiling-performance paradigm may therefore represent a valuable methodological framework for investigating movement optimization and mechanical loading strategies in other high-speed cyclic human activities.

Collectively, the present findings demonstrate that ceiling-tempo DU performance depends on a highly optimized coordinative strategy developed through long-term deliberate practice under extreme temporal constraints. The identified ceiling-level biomechanical benchmarks provide objective reference values for performance evaluation, movement retraining, and injury-prevention-oriented training design. From a translational perspective, the present ceiling-performance paradigm may offer a valuable framework for investigating movement optimization, mechanical loading management, and skill adaptation in other high-speed cyclic human movements.

## 5. Conclusions

Ceiling-tempo performance in world champions was characterized by explosive ankle-dominant control, temporally compressed movement execution, and precise rope–body coordination within extremely restricted temporal–spatial margins. These ceiling-level biomechanical benchmarks provide objective reference values for performance evaluation, movement retraining, and the design of training programs intended to support injury-risk management.

## Figures and Tables

**Table 1:** Participants’ general information (mean ± SD).

Group	N	Age (years)	Height (m)	Body Mass (kg)	Training (years)
WC	3	19.67 ± 2.08	1.69 ± 0.05	62.77 ± 9.93	11.33 ± 0.43
JNC	5	14.00 ± 2.83	1.55 ± 0.17	47.74 ± 15.08	5.33 ± 2.58

WC: world champion, JNC: junior national champion.

**Table 2:** Tempo-dependent motor control characteristics (average ± standard deviation) of selected key biomechanical markers in WCs.

Category	Parameters	120 DUs/min	140 DUs/min	Ceiling
Kinetic	Max vertical GRF (BW)	5.89 ± 0.34	5.92 ± 0.61	5.57 ± 0.23
	RFD (BW/s)	74.92 ± 8.45	92.26 ± 12.44	133.56 ± 13.54
	Vertical jump impulse (Ns/kg)	4.86 ± 0.19	4.26 ± 0.21	2.95 ± 0.37
Kinematic	Max COG height (% of BH)	68.08% ± 0.91%	67.26% ± 0.33%	56.04% ± 0.82%
	COG ROM (% of BH)	14.32% ± 1.22%	11.51% ± 0.74%	4.83% ± 1.31%
	Upper-limb control proxy (% of BH)	46.74% ± 1.49%	45.22% ± 0.91%	52.63% ± 1.04%
	Whole-body compression proxy (% of BH)	86.37% ± 3.92%	86.72% ± 1.43%	65.43% ± 1.01%
	1st–under clearance height (cm)	5.1 ± 0.7	4.1 ± 0.5	3.0 ± 0.4
	2nd–under clearance height (cm)	8.9 ± 1.1	8.4 ± 1.7	4.3 ± 1.0
	Highest rope mid-point (% of BH)	107.57% ± 1.46%	106.21% ± 0.68%	102.17% ± 0.58%
Time	Cycle time (ms)	500 ± 19	428 ± 7	255 ± 17
	Foot contact time (ms)	184 ± 14	145 ± 7	93 ± 4

DU: double under; RFD (BW/s): rate of force (GRF) development from zero to maximum normalized to body weight per second; Upper-limb control proxy (%BH): rope-handle lateral distance normalized to body height; Whole-body compression proxy (%BH): head-to-toe vertical distance normalized to body height.

**Table 3:** Tempo-dependent motor control characteristics (average ± standard deviation) of selected key biomechanical markers in JNCs.

Category	Parameters	120 DUs/min	140 DUs/min	Ceiling
Kinetic	Max vertical GRF (BW)	5.55 ± 0.34	5.58 ± 0.54	5.31 ± 0.19
	RFD (BW/s)	72.48 ± 5.39	90.46 ± 9.34	91.87 ± 7.95
	Vertical jump impulse (Ns/kg)	4.81 ± 0.25	4.19 ± 0.16	3.57 ± 0.23
Kinematic	Max COG height (% of BH)	68.54% ± 1.21%	67.47% ± 0.61%	65.44% ± 0.62%
	COG ROM (% of BH)	14.48% ± 1.04%	11.93% ± 1.84%	9.32% ± 3.51%
	Upper-limb control proxy (% of BH)	47.76% ± 4.34%	45.62% ± 1.63%	47.22% ± 0.64%
	Whole-body compression proxy (% of BH)	90.82% ± 1.81%	91.12% ± 1.32%	90.72% ± 1.36%
	1st–under clearance height (cm)	7.4 ± 0.9	5.5 ± 0.2	3.9 ± 0.6
	2nd–under clearance height (cm)	8.4 ± 0.9	8.2 ± 1.0	5.9 ± 0.4
	Highest rope mid-point (% of BH)	108.84% ± 1.42%	107.38% ± 0.84%	105.71% ± 1.02%
Time	Cycle time (ms)	499 ± 14	428 ± 11	362 ± 24
	Foot contact time (ms)	186 ± 16	149 ± 12	126 ± 8

**Table 4:** Between-group comparisons identified by *p*-values obtained from independent-samples *t*-tests with standardized effect sizes Cohen’s d (*p*-value, Cohen’s d). Results with *p* > 0.05 are not presented.

Parameter	120 DUs/min	140 DUs/min	Ceiling
Max vertical GRF	0.02, 0.68 *	0.02, 0.63 *	0.01, 1.11 **
RFD			0.00, 2.24 **
Vertical jump impulse			0.00, −2.17 **
Max COG height			0.00, −2.15 **
COG ROM			0.00, −0.94 **
Upper-limb control proxy			0.00, 0.85 **
Whole-body compression proxy	0.00, −1.23 **	0.00, −1.16 **	0.00, −8.32 **
1st–under clearance height	0.00, −2.68 **	0.00, −2.87 **	0.00, −0.89 **
2nd–under clearance height			0.00, −0.86 **
Highest rope mid-point	0.00, 0.70 **	0.00, 0.86 **	0.00, −13.13 **
Cycle time			0.00, −5.25 **
Foot contact time		0.02, 0.78 *	0.00, −4.70 **

*: significant (*p* ≤ 0.05), **: highly significant (*p* ≤ 0.01). Cohen’s d: positive values indicate higher values in WC, whereas negative values indicate higher values in JNC. Cohen’s d was interpreted as small (d ≥ 0.20), medium (d ≥ 0.50), and large (d ≥ 0.80).

**Table 5:** Statistical comparison results of within-group comparisons (*p*-value). Results with *p* > 0.05 are not presented.

Group	Parameters	120–140 DUs/min	140 DUs/min–Ceiling	Ceiling–120 DUs/min
WC	Max vertical GRF		0.02 *	0.03 *
	RFD	0.00 **	0.00 **	0.00 **
	Vertical jump impulse	0.00 **	0.00 **	0.00 **
	Max COG height		0.00 **	0.00 **
	COG ROM	0.00 **	0.00 **	0.00 **
	Upper-limb control proxy		0.00 **	0.00 **
	Whole-body compression proxy		0.00 **	0.00 **
	1st–under clearance height	0.00 **	0.00 **	0.00 **
	2nd–under clearance height		0.00 **	0.00 **
	Highest rope mid-point		0.00 **	0.00 **
	Cycle time	0.00 **	0.00 **	0.00 **
	Foot contact time	0.00 **	0.00 **	0.00 **
JNC	Max vertical GRF		0.02 *	0.02 *
	RFD	0.00 **		0.00 **
	Vertical jump impulse	0.00 **	0.00 **	0.00 **
	Max COG height		0.00 **	0.00 **
	COG ROM	0.00 **	0.00 **	0.00 **
	Upper-limb control proxy			
	Whole-body compression proxy			
	1st–under clearance height	0.00 **	0.00 **	0.00 **
	2nd–under clearance height		0.00 **	0.00 **
	Highest rope mid-point		0.00 **	0.00 **
	Cycle time	0.00 **	0.00 **	0.00 **
	Foot contact time	0.00 **	0.00 **	0.00 **

*: significant (*p* ≤ 0.05), **: highly significant (*p* ≤ 0.01).

## Data Availability

The data presented in this study are available on request and after appropriate IRB approvals.

## Abbreviations

The following abbreviations are used in this manuscript:

WC	World Champion
JNC	Junior National Champions
DU	Double Under
GRF	Ground Reaction Force
COG	Center of Gravity
ROM	Range of Motion
BW	Body Weight
BH	Body Height
RFD	Rate of Force Development
